# Does Having an Only Child Affect the Health and Well-Being of Older Adults?

**DOI:** 10.1093/geroni/igaf122.3294

**Published:** 2025-12-31

**Authors:** Madhyami Deshmukh, Peter Martin

**Affiliations:** Iowa State University, Ames, Iowa, United States; Iowa State University, Ames, Iowa, United States

## Abstract

Loneliness and depression significantly impact older adults’ health, well-being, and life satisfaction, but the role of gender and number of children remains unclear. This study examines how loneliness and depression affect life satisfaction and self-reported health, considering gender (male vs. female) and number of children (one vs. more than one) as moderators. Data from Wave 14 (2018) of the Health and Retirement Study (HRS) RAND Longitudinal Dataset were analysed, including 5,443 individuals (Mean age = 67.5). Bivariate correlations and blocked regressions assessed relationships, while moderation effects were tested using PROCESS MACRO in SPSS. Loneliness and depression negatively correlated with life satisfaction and self-reported health, indicating that individuals with higher loneliness and depression scores reported lower life satisfaction and poorer health. Blocked regressions confirmed that loneliness and depression significantly predicted both outcomes (β = -34, p < .001; β = -278, p < .001). Having more than one child positively influenced life satisfaction (β = .043, p < .001) after controlling for key variables. Gender moderated the relationship between loneliness and self-reported health, with the effect being stronger for females than males. These findings highlight the importance of family structure in aging and suggest that older adults with more than one child experience greater life satisfaction. This study informs future research on aging in single-child families and can guide policymakers in shaping family planning and aging policies.

